# A Malawi guideline for research study participant remuneration

**DOI:** 10.12688/wellcomeopenres.14668.2

**Published:** 2018-12-19

**Authors:** Stephen B. Gordon, Lameck Chinula, Ben Chilima, Victor Mwapasa, Sufia Dadabhai, Yohannie Mlombe, Malawi Research Ethics Workshop 2018 Participants

**Affiliations:** 1 Malawi LIverpool Wellcome Trust Clinical Research Program, Blantyre, Malawi; 2 University of North Carolina Research, University of North Carolina, Blantyre, Malawi; 3 National Health Sciences Research Committee, Lilongwe, Malawi; 4 Research Support Centre, University of Malawi College of Medicine, Blantyre, Malawi; 5 JHU Research Project, Johns Hopkins University, Blantyre, Malawi; 6 College of Medicine Research Ethics Committee, University of Malawi College of Medicine, Blantyre, Malawi

**Keywords:** Health research, remuneration, ethics, compensation, Malawi.

## Abstract

**Background: **Research participant remuneration has been variable and inconsistent world-wide for many years owing to uncertainty regarding best practice and a lack of written guidelines for investigators and research ethics committees.  Recent recommendations are that researchers and regulators should develop regionally appropriate written guidelines to define reasonable remuneration based on expense reimbursement, compensation for time and burden associated with participation.   Incentives to motivate participation are acceptable in specific circumstances.

**Methods: **We wished to develop regionally informed, precise and applicable guidelines in Malawi that might also be generally useful for African researchers and review committees.  We therefore reviewed the current literature and developed widely applicable and specific remuneration tables using acceptable and evidence-based payment rationales.

**Results: **There were good international guidelines and limited published regional guidelines.  There were published examples of best practice and sufficient material to suggest a structured remuneration table.  The rationale and method for the table were discussed at an inter-disciplinary workshop resulting in a reimbursement and compensation model with fixed rates.  Payment is recommended pro rata and equally across a study.

**Conclusions: **Transparent, fair remuneration of research participants is recommended by researchers and regulators in Malawi.  The means to achieve this are now presented in the Malawi research participant remuneration table.

## Introduction

Remuneration of participants in research by researchers is accepted practice ^1^. Further, the lack of any remuneration, particularly when research is conducted among vulnerable populations, is considered potentially exploitative and hence unethical ^2^. Regarding payments, clear statements from the Council for International Organisations of Medical Sciences (CIOMS) ^3^ as well as country-specific regulators such as the Office for Human Research Protection (OHRP) and the Federal Drug Administration (FDA) of the USA describe a code of practice where remuneration should not be sufficiently large to cause “coercion” or “undue influence” of participants ^1, 4^. Discussion of the means to determine appropriate remuneration in Africa without undue influence is the subject of this paper. There are currently no specific regional guidelines for Malawi.

## Methods

### Briefing paper and workshop discussion

We called together a workshop of Malawian research stakeholders to discuss the governance of research participant remuneration. The group was selected to include a wide range of stakeholders including government regulators (National Health Sciences Research Committee, Pharmacy Medicines and Poisons Board, Research Ethics Committees), Higher Education Institutions (University of Malawi College of Medicine, Malawi Polytechnic) research organisations (Malawi Liverpool Wellcome Trust, Blantyre Malaria Project, Dignitas) health care providers (Queen Elizabeth Central Hospital, District Health Officers) and other NGOs (Dignitas, Baobab). We included researchers with substantial first hand experience of participant qualitative research on this topic, but not research participants themselves.

To inform the workshop discussion, we carried out a broad literature review. Using the search terms “(volunteer or participant or patient) AND (remuneration or reimbursement or payment) AND (research)”, restricted to English published work in the period 1934–2018, we searched MEDLINE Complete, CINAHL Complete, Global Health and EBSCO host e-book collection. We found 24,605 hits and using the Relevance key, screened titles and brief summaries of the most relevant 200 hits. Then, we refined the search above by adding “AND (Africa)”. We found 424 hits and reviewed them all, citing only those specifically relevant to the discussion of research participant remuneration.

Before the workshop we circulated an early version of this document to define the appropriate contexts in which remuneration might occur and then framed the parameters for a Malawi remuneration table. We discussed in detail the need for transparent guidelines such that researchers could use a framework, and regulators could evaluate the rationale underpinning researcher protocols for remuneration ^3^.

### Malawi remuneration table development

Using the acceptable parameters obtained from the literature review, we determined a range of values for each parameter and then developed tables of values for each parameter. We then determined, in a public discussion, if these value estimates seemed reasonable to a participant group of researchers and regulators. Finally, we stated current exchange rates to allow comparison in other settings and after exchange rate changes. In this way, we produced a table that can be used by researchers and regulators alike to determine appropriate remuneration values.

## Results and discussion

### Literature review

***The development of current international practice*.** In 1998, research participant remuneration in the USA was shown to be inconsistent between different sites in multi-site studies, between studies at the same site and between procedures in the same study ^4^. This inconsistency was caused by anxiety about inducement and a lack of written guidelines for researchers and Research Ethics Committees (REC; also known as Institutional Review Boards (IRB), but REC will be used in this paper). Several studies indicated, however, that researchers were generally in favour of reimbursement both in clinical trials ^5^ and in the social sciences ^6^. The fact that remuneration might bias increased recruitment from impoverished participants was considered less of a problem by these researchers than alternatives such as no remuneration, compulsory participation or means-tested remuneration ^7^. Furthermore, studies indicate that payments did not cause volunteers to ignore risk ^8^, and that remunerated research participants considered a complex variety of factors before participation ^9, 10^. Phase 1 trial participants, in the USA, Belgium and Singapore were less likely to participate in trials of psychotropic drugs than other drug studies, and in trials with painful biopsy sampling compared to those with no pain involved ^11^. Further, adolescent patients in the USA made well argued decisions as well as tending to be altruistic compared to their parents ^12^. A study attempting to increase elderly patient participation in UK research showed that financial remuneration had little effect in reducing the disparity in participation between low income and high income areas with the former less likely to participate ^13^. Motivating factors reported by research participants in Malawi and Kenya often include community benefit, the advancement of science, personal interest and seeking additional health professional contact ^14– 16^. A Viewpoint paper in the *Lancet* in 2005 concurred that good regulator boards (REC) were critical in determining risk benefit ratio in research, and were more important in ensuring ethical research than the nature or scale of participant benefits ^2^. In modern day practice, the offer of payments to participate in studies causing harm, either by threat (a definition of “coercion”) or by an attractive offer (“undue influence”) has been outlawed by the appropriate processes of REC ^17^.

A 2018 framework to guide the ethical payment of research participants published in the *New England Journal of Medicine* ^1^, consistent with CIOMS guidelines ^3^, distinguished three rationales for remuneration which were:

- reimbursement of out-of-pocket expenses such as travel expenses,
- compensation for time lost at a rate approximately equal to unskilled labour, as the “work” of being a research participant is usually unskilled and
- some incentive to participation, calculated to improve the likelihood of study recruitment and completion ^1^.

Current practice is therefore that a minimum reimbursement is almost always expected, calculated as reimbursement for expenses and some compensation for time lost, albeit often not all the time lost, and certainly not the loss of potential earnings. The same guideline indicates that research participant remuneration should be prorated (paid per activity and not dependent on study completion), and clearly documented in both the REC protocol and patient information sheet. Recent detailed enquiry among investigators and REC Chairs in the USA, however, showed that only 19% of protocols included time-based compensation and only 12% of protocols included procedure-based estimation of burden ^18^. Incentives in the form of study completion bonus (9% of protocols) or increased attendance allowance (10% of protocols) were seldom included in REC documents. There was, however, good documentation of remuneration in the patient information sheet (94% of protocols) and this was usually prorated (73% protocols).

Current practice, as well as lacking transparency, is far from ideal as early career researchers particularly struggle to define appropriate patient involvement in research ^19^, and there is some evidence that remuneration is not adequate in some studies ^20^. We therefore concluded that transparent guidelines would be useful and applicable in Malawi, where the economic context of a low- or middle-income country (LMIC) drives particular concern about remuneration in research.

***The development of guidelines for remuneration in Africa*.** The underlying principles for remuneration in LMIC are identical to those elsewhere, but there are contextual considerations including culture that must be included in planning remuneration ^21^, and a relative lack of literature to guide researchers. A recent review has concluded that there is an urgent need for basic descriptive work in India (Marathe, 2018) but the KEMRI Wellcome group in Kenya have worked for several years to define types of volunteer remuneration and good ethical practice in Africa ^22, 23^. The KEMRI group have particularly pointed out that individual remuneration, including that offered to patients, is best served by financial compensation, but community recognition is best achieved with in-kind contributions to health facilities and community projects ^23^. Further, researchers should be aware of the potential for remuneration to produce family discord. There is a need for transparency in patient information sheets ^24^ and in actual practice ^25^ but nevertheless, guidelines can be developed in complex situations, including among patients with malnutrition ^26^, and among people living with HIV/AIDS (PLWHA). Among PLWHA, research participant remuneration has even been shown to increase patient’s well-being and self-worth ^27^ to the point where remuneration post-trial has been considered ^28^. It has been proposed that participant remuneration results in both individual and community good, and indeed that defining the remuneration only in terms of opportunity cost, matched to work, is to reduce the opportunity for community good ^29^.

In 2002 in South Africa, flat rates for research participation were suggested by the Medical Control Council but did not meet with community approval ^30, 31^. The South African NHREC (2012) guidelines for “Payment of Trial Participants in South Africa: Ethical Considerations for REC” note that a recommendation for a flat rate was made at a time when NHREC was not formally constituted, and the guidelines suggest that participants should be compensated for time, inconvenience and expenses ^32^. A recent Malawi recommendation of minimum rates for reimbursement when subjects attend facilities for research led to some confusion among researchers and REC, requiring discussion to understand the nuance and exceptions stated in the recommendation. In a study where the actual amount of reimbursement was discussed in Kenya, zero payments were determined to be unfair, and high reimbursement evoked suspicion (“what do they want if they are paying so much?”). Appropriate payment was related to the basic minimum wage ($3.50 per day) and to the amount a person might earn in the market selling goods ($80-300) ^16^. In a study of prospective participants in research bronchoscopy in Malawi, focus group discussion confirmed that remuneration for travel, food and lost earnings would be sufficient ^33^ and follow-up with these authors confirms that focus group discussion participants did in fact attend for multiple bronchoscopies

In conclusion, therefore, there are both *general* guidelines and examples of good practice internationally and in Africa. We could not find examples of *specific* guidance for researchers and regulators. Whilst investigators may be using appropriate formulae to determine research participant payments, they typically do not include the calculation in protocols submitted to REC wherever this has been audited. Given the lack of consistent guidelines, review committees are not able to make transparent judgments on the compensation offered to research participants ^20^. The situation in Malawi could be immediately improved by reference to current good practice and by publication of a specific guideline.

### Current practice and development of tables for Malawi research studies

There is a long tradition of both community- and hospital-based research in Malawi, and remuneration has been used in each of the three categories discussed above. As in other regions, financial remuneration is not the key motivational factor underpinning research participants involvement decisions in Malawi ^14^ and Kenya ^23^ — participant considerations of gaining access to otherwise inaccessible health care, examination and medical tests are equally if not more important ^33^.

***Reimbursement of expenses*.** Travel expenses on difficult journeys with food and accommodation costs are often paid to research subjects in Malawi ^14^. Where transport is difficult, involving multiple stages, crowded vehicles and long waits a private vehicle is sometimes provided as an alternative and is appreciated ^33^. In addition, young people and frail adults are usually accompanied on public transport. Telephone prepaid charge units are provided to study subjects or to community liaison volunteers when information is needed in surveillance studies ^34^.

Current advice is that actual travel and subsistence expenses should be reimbursed in studies but the practical means to do this is clumsy as receipts are often not issued and the administrative duty to make specific payments are often delegated to clinic staff. Accounting systems are very rudimentary and lack identifiable information. Further, *ad hominem* payments do not provide a basis for planning grant budgets and so the Malawi tables are constructed using reasonable predictive data. Travel was determined in three bands of <5 km, 5–10 km, and 10–15 km using standard minibus fares, but we recommend that a single rate be paid in any one study based on the radius of recruitment. At the workshop, participants contributed examples in region where variable travel reimbursement has led to confusion and frustration in the community from whom participants are recruited therefore this was noted and a recommendation included in the resulting table ( Table 1). Food was calculated using street restaurant food costs for mid-day meals and accommodation at rates charged by lodges with walled secure compounds. Overnight accommodation is very unusual in research studies, as is travel of more than 15 km.

**Table 1.** The Malawi research remuneration table – fill in the blank sections only to obtain the guideline total remuneration.

MALAWI RESEARCH PARTICIPANT REMUNERATION			
Reimburse Expenses	Rate in MK	Number of events	Total
a) Transport	*- see Note A*		
b) Subsistence (one meal)	1500		
c) Accommodation (one night)	15000		
**Compensation**			
a) Time *- see Note B*			
Total time in hours travelling			
Total time in hours at facility			
Time in days (day = 8hrs)	1000		
b) Burden *- see Note C*			
Procedure A	2000		
Procedure B	6000		
Procedure C	10000		
**TOTAL for study** *- see Note D*			

Note A: Transport rates Use the same for all participants in the study, based on furthest travel (5km = 300MK; 10km = 600MK; 15km = 900MK). If a travelling companion is required for minors or frail adults, double the sum allocated. Note B: Time. Use total time including travel, waiting, consultation, tests, waiting for results and treatment. Note C: Burden Categories. (A) mild discomfort: venesection <60ml, lung function testing, Xray = 2000MK. (B) Moderate discomfort: venesection >60ml, bone marrow, lumbar puncture = 6000MK. [C] Long or complex: bronchoscopy or GI endoscopy and biopsy/BAL = 10000MK. Note D: Minimum remuneration. Recommendation is that this should not be less than 7000 for studies attending facilities. *Divide Total by transport events (C3) to determine Average per visit (MK exchange rate at the time of writing 960 MK to 1 GBP; 700 MK to 1 USD).

***Compensation for time and burden*.** Particularly in healthy volunteer studies involving a procedure, compensation for both time and the burden of the study are paid. In the Malawi tables, the opportunity cost sustained by a volunteer has been calculated using the *total* time incurred in the study, to include travel to-and-from, waiting at the facility, direct involvement in the project and any time required to remain for observation after the process. Time used in completing follow-up diaries or on follow-up phone calls was also included. Time may be reimbursed as a time equivalent in minimum wage labour, even though this may under-estimate the opportunity cost ^16, 30^. We consider that the minimum wage provides a reasonable remuneration provided that time is adequately evaluated as participation in research is usually an unskilled task ^7^. In Kenya, reasons for participation included improve medical care, as published elsewhere ^14^. In a study requiring residential monitoring, participants cited saving for various projects (business, housing, school-fees) as a motivation to participate, and lack of family support as a disincentive ^16^.

The burden of participations in a study, including discomfort, anxiety or embarrassment was included in three bands of procedure discomfort. Risk *per se* should be minimised by study design and was not specifically reimbursed (this constitutes coercion); we took the view that any harm sustained should be covered by insurance. In the case of patients receiving a treatment or diagnostic test of proven value that might be outside the normal service, this would not constitute a burden. There are examples of good practice regarding research participant’s discomfort burden in Malawi. For example, for bronchoscopy studies, a consultative exercise including participants, research team and health care providers determined participant remuneration. Subsequently, participant interviews determined that most participants were content with the remuneration offered ^15^. Burden in terms of mental effort, concentration or fatigue afterwards (e.g. neurocognitive or ophthalmological examination) could be similarly in a graded manner by a process of consultation and evaluation.

***Incentives to participation*.** In Malawi, there is currently no equivalent of The Over-volunteering Prevention System (TOPS) found in other centres ^35^. Studies must therefore screen carefully and avoid the problem of over-recruitment by participant enquiry. One survey of nasopharyngeal carriage, offering a bottle of Coca-Cola as remuneration, observed a problem of over-recruitment (volunteers attempting to participate twice) and had to re-structure consent and sampling processes ^36^. Given that a risk of over-volunteering therefore already exists in Malawi, and that there is no surveillance system in place, caution is advised on offering incentives to participation.

In the tables, we noted that remuneration of participants should be pro-rated (in the sense of *pro rata*, in that completion is not necessary) because of fairness needed when participants are *required* to withdraw from a study (e.g. owing to side effects in a drug trial). Participants might fail to disclose adverse side effects if this would sustain a reimbursement penalty. Completion bonuses run a risk of participant coercion. Increased payments for repeat procedures that become tedious and burdensome were discussed as an option but there was no consensus of support for this approach as the principles of reimbursement and compensation were held as more important than estimates of boredom.

### Malawi remuneration tables

In order that the challenge of presenting clear, specific guidelines that would allow transparency in reviewing protocols at REC, the Malawi research remuneration tables were developed as shown in Table 1 and Table 2. Table 1 shows the table for calculation, with Table 2 completed for a simple study and Table 3 for a more complex study. Some degree of international comparison may be achieved using the dollar exchange rate provided. This does not, however, take any account of purchasing power or local wages therefore appropriate regional and international comparison will be made by replacing the values for round-trip travel (5, 10 and 15km) and for a day’s wages at the local minimum wage. An active spreadsheet is provided in Supplementary File 1.

**Table 2.** Worked example for a simple study. Volunteers attend for collection of a complex data set in which the process of travel (2 hours) and study completion (4 hours) takes 6 hours including a large volume blood test. They re-attend on a second occasion taking 2 hours of travel as before but only 2 hours of time at facility with a smaller blood test. This example using the Malawi table shows the remuneration for a participant who travelled 15km round trip (900MK), was provided lunch (1500MK) in a day that involved 6 hours of attendance and one large blood sample and was then followed up on a second visit which required 2 hrs time in travel and 2 hours at the facility (hence 10 hours total) and a small blood sample. Remuneration was MK12550. The US dollar exchange rate was 719 MK to the dollar.

MALAWI RESEARCH PARTICIPANT REMUNERATION			
Reimburse Expenses	Rate in MK	Number of events	Total
a) Transport - see Note A	900	2	1800
b) Subsistence (one meal)	1500	1	1500
c) Accommodation (one night)	15000	0	0
**Compensation**			
a) Time - see Note B			
Total time in hours travelling		4	
Total time in hours at facility		6	
Time in days (day = 8hrs)	1000	1.25	1250
b) Burden - see Note C			0
Procedure A	2000	1	2000
Procedure B	6000	1	6000
Procedure C	10000	0	0
**TOTAL for study**- see Note D			**12550**

**Table 3.** Worked example for a complex study. Volunteers attend for collection of a complex data set 4 hours and a large blood test. They re-attend 6 further occasions taking one hour and a simple blood test each time. There are 2 additional visits for bronchoscopy (4 hours). This example shows the participant travelled 15km round trip, attended for 4 hrs and had a large blood test, then 6 follow-up visits and 2 visits for bronchoscopy resulting in a total study remuneration of MK 55100.

MALAWI RESEARCH PARTICIPANT REMUNERATION			
Reimburse Expenses	Rate in MK	Number of events	Total
a) Transport - see Note A	900	9	8100
b) Subsistence (one meal)	1500	3	4500
c) Accommodation (one night)	15000	0	0
**Compensation**			
a) Time - see Note B			
Total time in hours travelling		18	
Total time in hours at facility		18	
Time in days (day = 8hrs)	1000	4.5	4500
b) Burden - see Note C			0
Procedure A	2000	6	12000
Procedure B	6000	1	6000
Procedure C	10000	2	20000
**TOTAL for study**- see Note D			**55100**

## Conclusion

International guidelines and current best practice both indicate that structured remuneration of research participants is ethical and appropriate in Malawi. From a review of the literature, we provide an underpinning rationale for remuneration based on reimbursement of expenses and compensation for time and burden, but not incentive to participate. We then provide specific tables to guide researchers and regulators in the amount to remunerate. We publish these in Wellcome Open Research in order that revised versions can be updated and available as required.

## Data availability

All data underlying the results are available as part of the article and no additional source data are required.

## Supplementary material

**Supplementary File 1. Working examples of the remuneration tables shown in Tables 1–3.**
