# Supplementary material for: A Malawi guideline for research study participant remuneration
Source: Wellcome Open Res. 2018 Dec 19;3:141. Originally published 2018 Nov 1. [Version 2] doi: 10.12688/wellcomeopenres.14668.2 (PMC6329041; doi:10.12688/wellcomeopenres.14668.2)
Supplement: Peer review history

# Peer review: Referee response for version 2

**DOI:** 10.21956/wellcomeopenres.16342.r34475

**Published:** 2019-01-10

**Authors:** Melissa Gladstone

**Affiliations:** 1 Department of Women’s and Children’s Health, Institute of Translational Medicine, University of Liverpool, Liverpool, UK

I am grateful to re-review this paper and feel that the team have addressed some of the concerns raised. It is difficult to see exactly where the changes have been made as they are not highlighted in the text but some information is provided in the rebuttal comments box. 

 As the team highlight that there has been previous qualitative work done by community participants and has referenced this, it might have been helpful to delineate this with a little more information and explained therefore why the stakeholder group that was chosen for this work was chosen with this in mind. I agree with reviewer 1 that including some "next steps" in terms of qualitative work which could be done with the community relating to this topic would be important as i still feel that this was a gap in the research conducted for this report. 

 The Wellcome Open Research platform will be a forum where this can be updated and in doing so, feedback and evaluation of the proposed system would be important to provide. Delineating this within the discussion and conclusions might be helpful for the reader in order to know what to expect in the future. 

 I think it is important that this article is indexed to enable further debate and discussion but hope that the team will make it clear that this is why they have published the work at the stage it is. 

 Note - Marathe 2018 is in () within the text but not referenced formally.

I have read this submission. I believe that I have an appropriate level of expertise to confirm that it is of an acceptable scientific standard.

# Peer review: Referee response for version 2

**DOI:** 10.21956/wellcomeopenres.16342.r34476

**Published:** 2018-12-20

**Authors:** Heather Draper

**Affiliations:** 1 Department of Social Science and Systems in Health, University of Warwick, Coventry, UK

I confess that am disappointed with the authors’ responses, which have been minimal throughout. The response to the reviewers did not highlight where and what changes were made leaving me to compare the two versions of the paper to find the revisions.

 The authors seem to have elected not to elaborate on weight-bearing assumptions like the one related to coercion, for example. There a substantial literature on the balance to be struck between payment-related coercion and non/under-payment-related exploitation that the authors could have drawn on to refine their understanding of coercion. They have also not addressed the question of the disparity that may arise from averaging payments and which is clear from their two worked examples (removing the average from table two only masks rather than addresses the issue). The reason given for not including research participants/lay members was because this was a project about governance issues. This seems a thin response (especially given their own reluctance to tackle meaty governance concerns) and they make no suggestions of future work that could usefully be done to e.g. address questions – like the averaging if payments – with those who would be affected, even if the authors are not able to do this work themselves. Some relatively simple fixes (like removing the confusing pale grey from the table) have been ignored completely. In other places the minimal approach to addressing comments have led to new and confusing sentences.

 For example:

 The relevance of this sentence needs to be explained:

 In Kenya, reasons for participation included improve medical care, as published elsewhere14. In a study requiring residential monitoring, participants cited saving for various projects (business, housing, school-fees) as a motivation to participate, and lack of family support as a disincentive1.

 This sentence doesn’t make sense:

 In the case of patients receiving a treatment or diagnostic test of proven value that might be outside the normal service, this would not constitute a burden.

 In other cases (e.g. my observation in relation to the difficulties of balancing engaging robust and transparent accounting help with participant confidentiality) have been addressed by adding out of place sounding sentences, which tick of box of gesturing towards addressing comments without actually doing so.

 In conclusion, I do not want to stand in the way of having the paper listed for indexing (because the issue is an important one that needs to be aired) but I think that it could have been a much better paper if the authors had addressed the reviewers’ comments more robustly and with greater enthusiasm.

I have read this submission. I believe that I have an appropriate level of expertise to confirm that it is of an acceptable scientific standard.

# Peer review: Referee response for version 1

**DOI:** 10.21956/wellcomeopenres.15970.r34179

**Published:** 2018-11-30

**Authors:** Melissa Gladstone

**Affiliations:** 1 Department of Women’s and Children’s Health, Institute of Translational Medicine, University of Liverpool, Liverpool, UK

This is a descriptive paper demonstrating the findings from a literature review on renumeration in research specifically targeted on papers from African settings as well as the findings from a research ethics workshop conducted in Malawi.

 The paper is an important one as it brings up a discussion in relation to the need for clearer guidelines with regards to remuneration for research which is a particularly difficult subject in low income settings such as Malawi where there are often concerns about coercion. It opens up a discussion which is really important and provides a pragmatic approach to addressing it.

 The results of the review were interesting but it was not always clear whether the results were specifically of those articles found from African settings or a mixture of articles that were found and felt to be relevant. There was not much critical appraisal of these articles but maybe not that necessary for this paper. It might have been helpful to have a list of those papers found from Africa in a supplementary file for those who would like to look into this in more detail.

 The paper describes the guidelines for remuneration as having been developed mainly from a research ethics workshop conducted in Malawi. Although the list of authors is at the end of the paper, it might have been helpful to have a list of the professions of those persons to understand the backgrounds of those participating. There was no representation from lay persons and the group who were involved were very research-focussed as far as I can gather. It would be important to discuss this in the limitations and to think through how this might be moved forward in the future to ensure that the points of view of research participants, lay persons and communities are considered. It will be helpful to know what the cost is to families or single individuals and whether this varies by gender and profession. I agree that not enough reference is made to consider compensation for those who might be accompanying a minor or a person with major health issues who needs support. This needs to be discussed further or considered in future papers or research.

 It is not clear what “burden” means and whether there again should be some future consideration of how this should be classified. Some studies may not take bloods but may do a number of complex investigations or tests which take a large amount of time and require the individual to be motivated and engaged. There was some discussion about the time factor taken but it was not clear to me as to how one would distinguish between someone who might be having a large amount of blood taken but not be there for very long vs someone who might be having complex hearing or vision testing or detailed neuromotor examinations which might take hours to complete. Maybe this is something that is a limitation and which needs further discussion for future work.

 This paper should however be indexed to widen the debate and to put something forward for discussion but with some of these limitations taken into account.

I have read this submission. I believe that I have an appropriate level of expertise to confirm that it is of an acceptable scientific standard, however I have significant reservations, as outlined above.

# Response

**Published:** 2018-12-12

**Authors:** Stephen Gordon

**Affiliations:** Malawi Liverpool Wellcome Trust Programme of Clinical Tropical Research, Malawi

We thank Dr Gladstone for thoughtful and helpful comments made on the first version.

We have improved the clarity and purpose of this paper by responding to all the points raised (more than 10) but in particular:

1. We thank Dr Gladstone for identifying the usefulness of this work.
2. We have clarified each point where clarification was requested  including the composition of the workshop attendee list, decisions around remuneration of expenses (e.g. travelling companions).
3. We have discussed the group experience with working with research participants in this field.  We did not have representation at the stakeholder workshop as the governance of remuneration was the focus rather than the financial amount.  6 publications of studies researching participant responses were cited, with authors from 3 of them present at the workshop.
4. We have not provided a list of papers cited/searched but we have checked the method described and found it to be robust on several platforms.  We think that a current search is quick and easy and will generate hits in an up-to-date manner.
5. We thank Dr Gladstone for raising the point about neurocognitive and other fatigue burdens in research participation.  The issue of “burden” is complex and study-specific.  We think that the guideline table allows investigators to work with the concept of a range of burden in research, and to propose acceptable remuneration for discomfort, be it physical, mental or other.

Once again, we thank the reviewers and offer this much improved manuscript for indexing.

# Peer review: Referee response for version 1

**DOI:** 10.21956/wellcomeopenres.15970.r34175

**Published:** 2018-11-22

**Authors:** Heather Draper

**Affiliations:** 1 Department of Social Science and Systems in Health, University of Warwick, Coventry, UK

I am not able to answer the clinical practice article-specific questions because this paper does not fit neatly into this category (though it is not obvious where it would fit any better and one should not be deterred from accepting it on that ground alone).

 This paper makes a useful addition to the literature. It publishes (for comment/benefit of others in the same position) a method (in the form of a table) of making a transparent calculation of reimbursement for research participants in Malawi. This takes into account immediate out of pocket expenses (such a travel and subsistence), time taken to participate – including travel time (opportunity costs to the participant, calculated on the basis of the minimum wage) and recompense for burden (using three categories with different sums according to severity). The total is then averaged accordingly to the number of participation events. The authors arrived at the table by a combination of literature review and consultation with researchers and regulators.

 I have annotated a copy of the paper for the authors, which can be viewed here. What follows is a summary of the main points of concern.

- The paper would be improved if the authors provided a more robust and detailed justification of: their definition of coercion; decision to go with minimum wage; the obligation on RECs to ask how calculations have been arrived at if an explanation is not provided; the categories of burden adopted (e.g. is a large blood test really equal to a lumber puncture?); lack of consensus around repeat payments for multiple tedious visits.
- Include the summary document referred to on page one in the supplementary materials.
- The consultation/discussion/participant group appears not to have included lay people. Why was this? Do the authors consider that this is a significant limitation? Either way this should be discussed. Are there plans to evaluate participants’ reaction to the assumptions and resulting calculations in the table?
- The authors state on page 4 that in Malawi expenses for a companion are also often paid. Why is this and why is this not reflected in the table (how did you decide to exclude this)?
- The decision to average the payments needs greater elaboration and justification. Note, for example, that the average for participation in the first worked example is more than in the second (which is considerably more onerous). Also note that in the second worked example a participant might not seem to have been fairly remunerated (according the amounts proposed by the authors as fair) if she was withdrawn by the researchers after e.g. the first visit and first bronchoscopy.
- The calculations in the first worked example don’t make sense to me. On the basis of the notes provided, the total amount of time spent in the study appears to be 9 hours 2 x 2=4 hours travelling + 1 x 4 hour appointment + 1 x 1 hour appointment.
- In the same table, it was also not clear why any allowance for burden was being made if the patients were being brought back to be given results. The authors previously state on p. 5 that  'diagnostic test of proven value ...  would not constitute a burden'? This needs to be clarified in the notes or changed.

 

 More minor points:

- When describing the literature it may be useful to record where the research was carried out and to distinguish between papers that report empirical findings and more philosophical pieces where authors are arguing for their own positions. Literature seems to be taken at face value with no discussion of validity. Consider whether literature pertaining to one sort of research (e.g. phase I trials) is pertinent to others (e.g. phase III trials).
- Be cautious of over generalizing – e.g. it is not obvious that current practice is to include expenses AND compensation for time lost. Current practice where?
- The pale grey fill on Table 1 is confusing. Consider removing. Consider modifying the presentation of the table so that the headings are in pale grey – in the worked examples alternate lines are pale grey.
- Last sentence first column on page 5 unclear.
- The supplementary file does not add anything and could be deleted.
- Other minor points/comments included in the annotation, including typos where spotted.

I have read this submission. I believe that I have an appropriate level of expertise to confirm that it is of an acceptable scientific standard, however I have significant reservations, as outlined above.

# Response

**Published:** 2018-12-11

**Authors:** Stephen Gordon

**Affiliations:** Malawi Liverpool Wellcome Trust Programme of Clinical Tropical Research, Malawi

We thank Professor Draper for thoughtful and helpful comments made on the first version.

We have improved the clarity and purpose of this paper by responding to all the points raised (more than 40) but in particular:

1. We thank Prof Draper for identifying the usefulness of this work.
2. We have clarified each point where clarification was requested including the definition of coercion, the composition of the workshop attendee list, decisions around remuneration of expenses (e.g. travelling companions).
3. We have discussed the group experience with working with research participants in this field.  We did not have representation at the stakeholder workshop as the governance of remuneration was the focus rather than the financial amount.  6 publications of studies researching participant responses were cited, with authors from 3 of them present at the workshop.
4. We have corrected the (embarrassing) error in our worked example and the legend.  This occurred with a mistake in combining 2 previous examples.
5. We have identified the country of origin of source material where missing, as much of the discussion is context specific.
6. The issue of “burden” is complex and study-specific.  We think that the guideline table allows investigators to work with the concept of a range of burden in research, and to propose acceptable remuneration for discomfort, be it physical, mental or other.

Once again, we thank the reviewers and offer this much improved manuscript for indexing.
